# Machine-Learning-Based Human Fall Detection Using Contact- and Noncontact-Based Sensors

**DOI:** 10.1155/2022/9626170

**Published:** 2022-09-06

**Authors:** Ayush Chandak, Nitin Chaturvedi, Dhiraj

**Affiliations:** ^1^BITS Pilani, Pilani Campus, Pilani, India; ^2^CSIR-CEERI, Pilani, Rajasthan, India

## Abstract

Automated human fall detection is an essential area of research due to its health implications in day-to-day life. Detecting and timely reporting of human falls may lead to saving human life. In this paper, fall detection has been targeted using machine-learning-based approaches from two perspectives regarding data sources, that is, contact-based and noncontact-based sensors. In both of these cases, various methods based on deep learning and machine learning techniques have been attempted, and their performances were compared. The approaches analyze data in fixed time windows and extract features in the time domain or spatial domain which obtain relative information between consecutive data samples. After experimentation, it was found that the proposed noncontact-based sensor techniques outperformed the contact-based sensor techniques by a margin of 1.82%. After this, it was also found that the noncontact-based sensor techniques outperformed the state of the art of noncontact-based sensor results by a margin of 3.15%. To better suit these techniques for real-world applications, embedded board implementation and privacy preservation of subject by using advanced methods such as compressive sensing and feature encoding need to be attempted.

## 1. Introduction

The falls are widespread among elderly individuals due to the weakening of body parts which occurs due to aging [1]. Sudden falls can affect not only the elderly but also people of all age groups. Falls can cause injuries ranging from fractures to concussions and, in extreme cases, even death. Due to this, falls have recently been an extensively researched topic, and various automation methods are being studied to detect and analyze them. Thus, there is a growing need to use the latest techniques for the automated detection of falls.

Falls can occur in various different ways. Oneill et al., in their study, classified fall into forward, sideward, and backward [2]. Out of this, it was observed that forward fall was the most prominent fall, with 56.33% of all the falls, with 17.60% of falls being backward and 23.23% of falls being sideward.

Also, the chances of falls increase with increasing age varying from about 26% of people suffering falls at least once a year in the age group of 65 to 74 to 36.5% in the age group above 85 [3]. Thus, detecting falls among the elderly is more critical due to increased chances of falls and slow recovery rates. The reasons for falls among the elderly can be many, and the consequences can be severe if not detected in time. Unconsciousness resulting from the fall can delay the treatment process; thus, an automated fall detection system can help the concerned people know about the fall quickly. For this purpose, falls can be detected through either contact-based or noncontact-based sensors. Both methods are equally important in different ways.

There are many methods through which fall detection can be approached depending on which sensors are used, like accelerometers, gyroscopes, cameras, and others. In this paper, we have discussed some ways to detect falls using vision-based tools and contact-based sensors. Cameras are becoming very prominent in everyday environments and can be found in various environments such as railways and airports. At the same time, accelerometers are the devices used for capturing acceleration. When fitted to a body part, they provide information on the acceleration of the body parts. Using the information from data captured on acceleration and the change in acceleration, detection algorithms can be designed and trained to perform automatic fall detection. Thus, these two cases provide very different approaches to fall detection.

Neural networks are becoming very popular in the areas of fall detection due to increased efficiency in the detection process. Convolution neural network (CNN) is the method that specializes in analyzing sequential and visual data [4]. CNNs have been used for tasks such as image classification, natural language processing, and human activity classification. Thus, in this paper, many proposed approaches are designed based on neural networks architectures.

This work has contributed towards (1) detecting falls based on cameras and (2) detecting falls based on smartphone sensors. Various approaches are applied and tested in both cases. Then comparisons of these approaches are presented. Some techniques are also introduced to help reduce computation time and efficiency.

The paper first provides related works in the field and the description of the detection dataset used (UP-Fall). Then, the approaches used for contact-based and noncontact-based fall detection are discussed, followed by the results and analysis. Then, finally, conclusions are drawn.

## 2. Related Works

Recently, in the field of human activity recognition, many research studies are being done as this is an essential aspect and has a wide variety of use-cases such as healthcare, sports, and elderly care. Of the research works done, many utilized a variety of ways to detect falls, like using classical ML models, which include Support Vector Machine (SVM), K-nearest neighbors, and Random Forest, and neural networks based model. Recently deep learning models have become very popular due to their promising results.

The falls systems were mainly based on contact-based and vision-based sensors. Thakur [5], in his research, analyzed various recent fall detection systems using contact-based, ambiance-based, and vision-based sensors. Many datasets are also increasingly becoming available in both aspects. The two following domains were analyzed for the work being done in automated human fall detection.

### 2.1. Machine-Learning-Based Fall Detection Using Contact Sensors

This type of sensor relies on sensors being fitted on the subject to detect any sudden motion indicating a fall. They include using sensors such as accelerometers and gyroscopes. These sensors are generally fitted on the watch or mobile phone. These sensors record acceleration of the body parts on which they are fitted. The change in acceleration due to change in the activity changes acceleration is utilized for fall detection.

Many different approaches in this domain have been applied and researched utilizing different aspects. Khojasteh et al. [6], in their research, proposed a fall detection system based on wearable devices like smart wristbands where they utilized a threshold-based solution for this purpose in which if any change in acceleration crossed a certain threshold, then fall signal was generated. Kwolek [7] utilized a similar method based on threshold-based detection on accelerometers. He proposed an innovative solution based on a fuzzy-based system to authenticate the fall event. Şengül et al. [8] used a quaternion algorithm to detect falls and send a signal to the concerned people with the patient location. More recently, fall detection using convolutional neural networks has become popular due to these networks' ability to learn features without any data processing and their robustness and flexibility. Santos et al. [9], in their research, utilized CNN-based models on the accelerometers-based dataset.

### 2.2. Machine-Learning-Based Fall Detection Using Noncontact Vision Sensors

The major advantage of this type of fall detection is that the user is not required to wear any type of sensor. However, the user is required to be always inside the region of operation of the camera. Also, detecting falls on the border is difficult in these cases [10]. They include the detection of falls using video camera recordings, I.R. sensors, bioradars, and others.

Many different approaches have been attempted in this case utilizing machine learning and deep learning techniques, as well as using different preprocessing techniques like optical flow images, image lightning, and others. Rougier et al. [11], in their study for detecting fall events using a video surveillance camera, used the shape deformation of humans in the video frames during the fall for the detection. Furthermore, shape analysis models were used for training. Capturing human deformation during the fall is a good technique as it is very much noticeable in the fall video frames. Anishchenko et al. [12], in their research, showed the advantages of using bioradar's systems to detect human falls. The research used wavelet transform and neural networks for fall detection. At the same time, Miao et al. [13] utilized CNN-based models to detect human-based activity and further modified them for detecting falls. In a similar approach, the CNN model using 3D ConvNets was approached in this case as well. More recently, latent feature polling was used for detecting falls effectively in ADLs (activities of daily living) [14].

## 3. Dataset Description

**Figure 1:**
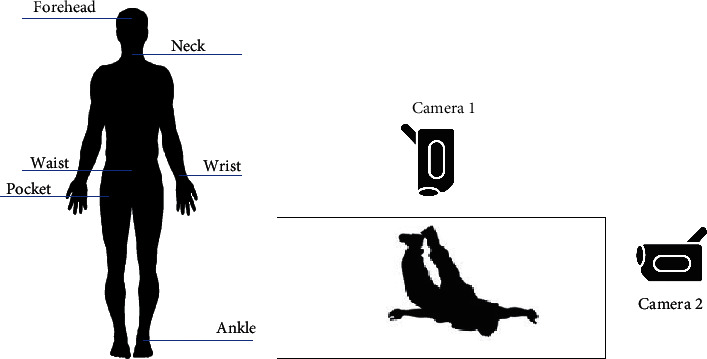
Position of the sensors. (a) Contact-based sensors located on the human body. (b) Video camera location layout for the lateral and frontal views.

For this research, we have chosen a publicly available dataset, the UP-Fall detection dataset [15], after analyzing the various fall detection datasets [16–20]. Due to variability in subjects, activities, and different lighting conditions, this dataset provided real-life conditions for training and testing. Of all the datasets, the UP-Fall dataset provided more variability in terms of subjects, activities, frontal and lateral views, a greater number of trials, and so forth, and it contained a larger number of videos enough for effectively training the network. Besides that, it also provided data from contact-based sensors like accelerometers, gyroscopes, and others, which can also be used for trying other approaches to fall detection. The dataset and features are publicly available [21]. The dataset consists of 11 activities performed by 17 young, healthy individuals (8 females and 9 males) in the age group of 18–24 years. Out of the 11 activities performed, 6 consisted of daily human activities, which are lying, jumping, picking up an object, sitting, standing, and walking, and 5 consisted of fall activities, out of which two consisted of falling forward using a hand and knees, two consisted of falling sideways and backward, and one consisted of falling sitting on a chair.  Figure 1 describes the positions of some of the contact-based and noncontact-based sensors that were used in the UP-Fall dataset.

For the purpose of the experiment, we have used data recorded at 18 FPS from the two cameras and Inertial Measurement Unit (IMU) sensors fitted on the wrist and right pocket of the subject. We aim to implement a system to distinguish between fall and nonfall activities using data from contact-based sensors such as accelerometers as well as noncontact-based sensors such as cameras.

## 4. Methodology and Implementation

**Figure 2:**
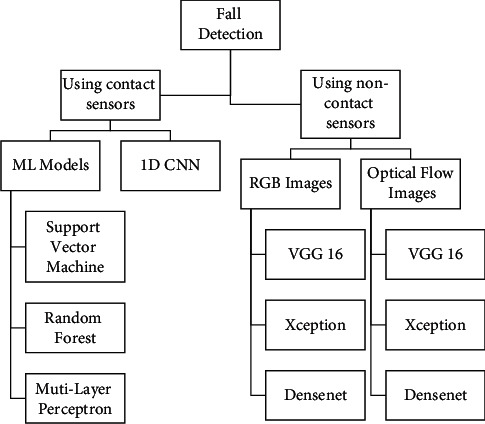
Overview of the methods and architecture used in the paper.

Human fall detection can be captured through a variety of methods. The analyzed methods were chosen such that they are not much affected by the environmental factors and can be applied to any general setting (not requiring any special arrangements). The following section describes the methods and the architectures that were implemented for contact-based and noncontact-based fall detection (Figure 2).

### 4.1. Detection Using Contact-Based Sensors

Data from the triaxial accelerometer was utilized in this case for fall and nonfall prediction. Two 3-axial accelerometer sensors were fitted on the subjects in the right pocket and on the right wrist simulating the wristwatch and cell phone, respectively. Since the accelerometers are kept very close to the subject, predicting the fall using them is very institutive for automated detection. Machine learning architectures and a 1D CNN-based approach were deployed for this purpose.

**Figure 3:**
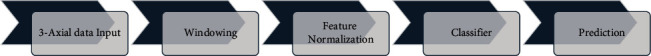
The process outline used for fall detection using contact-based sensors: 3-axial data input from accelerometers is processed and then passed through the classifier, which identifies whether there is a fall or not.

Firstly, the 3-axial data received from the accelerometers was trimmed to 3 seconds in order to capture the fall and nonfall parts effectively. To standardize the dataset, the data points were subtracted from the mean and divided by the standard deviation. Afterward, continuous 1.5 seconds of fixed arrays were drawn from the normalized data, which were then appended one after the other in 3rd dimension order. The obtained flow stacks were passed through the classifier (ML models and 1D CNN) to predict the fall event. The overall process outline can be seen in Figure 3. For the experiments, the dataset was divided into 70% for training and 30% for testing.

#### 4.1.1. Machine Learning Methods

In this paper, the following machine learning classification methods were implemented for the detection:

1. Support Vector Machine (SVM): This method differentiates between the classes by creating a hyperplane and mapping the inputs on it. The hyperplane is further optimized by training and then acts as a decision boundary for prediction. The target of the algorithm is to maximize the gap between the data points and the hyperplane. SVM is a very popular ML algorithm and is used extensively in fall detection systems [22].
2. Random Forest (RF): This method is a widely used machine learning algorithm that works on the principle of ensemble learning. It works by creating an ensemble of decision trees through which the input is passed, and, on the basis of the mode of the responses received, the output of the algorithm is computed. The greater number of trees leads to better accuracy and reduces risks of overfitting [23].
3. Multilayer Perceptron (MLP): This method is a feedforward method passing inputs through the multiple layers before computing the final output. Each node has a neuron that utilizes a nonlinear activation function. It uses the back-propagation technique for the training of the model [24].

The parameters used for the machine learning architecture used are described in Table 1.

#### 4.1.2. 1D CNN Network

CNN is very good for classifying sequential data. Thus the data from the 3-axial accelerometers is a good match for applying CNN-related methods. Lee et al. [25], in their research, analyzed the benefits of using the 1D CNN process for fall detection. In the research, 1D CNN was shown to be better than most of the traditional ML methods when using time-sequential data.

**Figure 4:**
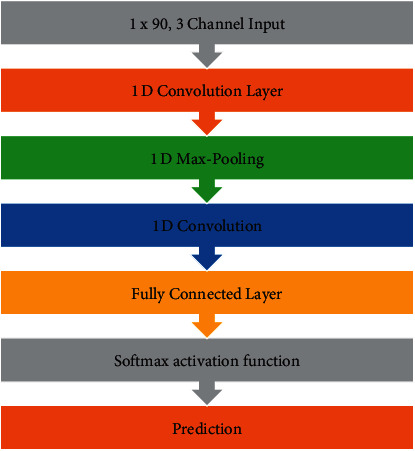
Architecture used in case of 1D CNN for sensor-based training of the model.

The convolutional and pooling layers used in 1D CNN are one-dimensional with height and width of 1. The algorithm that was used for this purpose is described in Figure 4. It is a simple convolution method with 1st layer being convolutional followed by max pooling and then one more convolutional layer followed by a softmax activation function for calculating probabilities. The network was trained for 500 epochs.

### 4.2. Detection Using Noncontact-Based Sensors

**Figure 5:**
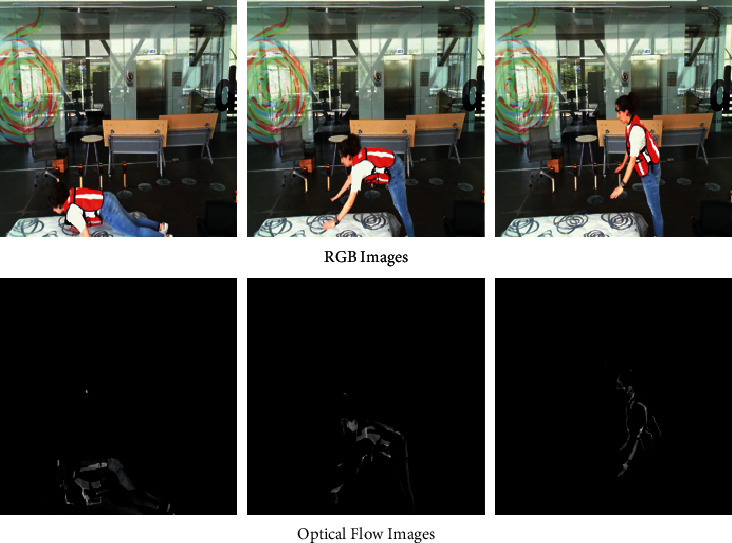
RGB and its optical flow image equivalent. Optical flow images help in removing background noises and capture only the motion.

**Figure 6:**
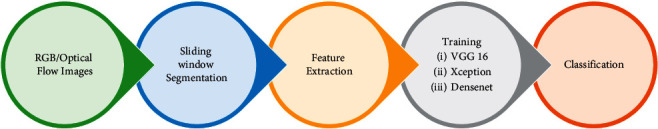
Process pipeline used for fall detection using contact-based sensor.

RGB camera recordings captured from the frontal and the lateral side of the subject were utilized in this method for predicting the fall and nonfall events. Additionally, the RGB images were also converted to optical flow images. Optical flow images are useful in removing the background noises as they only capture the motion between the consecutive frames, as shown in Figure 5. Both RGB and optical flow images were attempted for the detection.  Figure 6 describes the stepwise procedure that was used for training the dataset. Overall, it consisted of 5 steps which were dataset collection, segmentation, feature extraction, training of the model, and classification. These steps are explained in the following sections.

#### 4.2.1. Dataset Used

Camera recordings from the UP-Fall detection dataset were used as the data input. Videos were captured from the frontal and lateral sides of the subject at 18 FPS. It comprised 17 subjects performing 11 activities in three trials, as shown in Table 2. Out of the 11 activities, 5 were classified into fall and 6 into nonfall category.

To keep the computational costs low, the resolution of the images was reduced to 224 × 224 from 640 × 480. Moreover, the recordings were trimmed to 50 frames each, given that 50 frames are sufficient for identifying any fall or nonfall event. The dataset was further divided into training set and testing set in an 80 : 20 ratio.

#### 4.2.2. Segmentation

**Figure 7:**
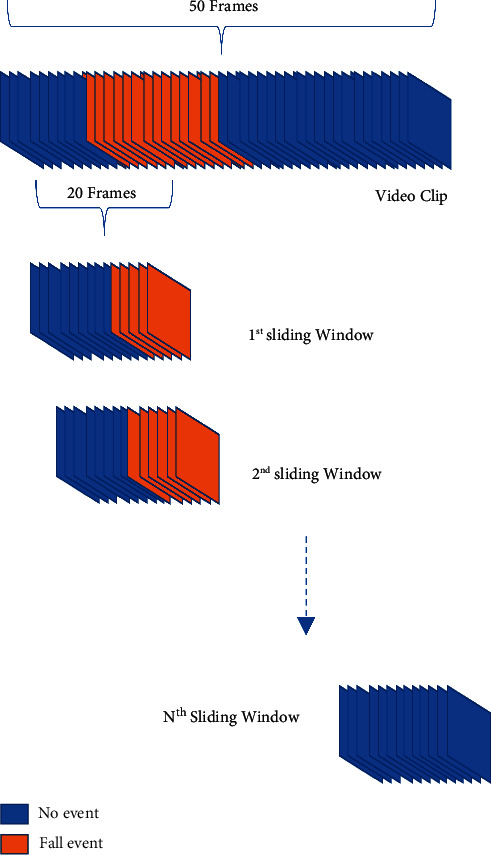
Sliding window technique used to capture stacks of consecutive frames. Here frames in orange color are the ones that contain events important for the detection.

As observed from the dataset, detecting any fall event does not require more than 20 frames. Using this observation, the sliding window segmentation technique was attempted to capture video frames containing only the fall event, if present. So, out of the *N* frames, if *L* is the length of the window used, then using a sliding window of 1 frame, *N* − *L* + 1 windows were captured, as shown in Figure 7.

#### 4.2.3. Feature Extraction

The target of this step is to get the most important features that are relevant to the experiment. This part involves removing the noise from the data and capturing the part that is most relevant to the training of the architecture. The noise can be any repetitive data or unwanted data points that can affect the convergence of the model.

For vision-based approaches, the optical flow method delivers good information for clear movements between the images and removing any background noises [26]. Thus, for capturing the motion and removing the background noises, the effect on the model by converting the RGB images to optical flow images was analyzed. For this purpose, out of 50 frames, the difference in the intensity of consecutive frames was taken. Thus, starting from the 1st frame, the difference between the 1st and 2nd was taken, followed by that between the 2nd and 3rd, and so on till the 49th and 50th frame. Thus, in this way, we obtained 49 optical images representing the activity.  Figure 6 describes the effect of converting an RGB image to an optical flow image in the case of the UP-Fall dataset.

#### 4.2.4. Model and Training

For training the model with video recordings as the input, 3D convolutional neural networks offer the capability to capture spatial information in time by stacking consecutive video frames. Tran et al. [27], in their study, analyzed the benefits of using 3D ConvNets when using video datasets for training. By using stacks of consecutive frames as the input, they allow capturing the features that take the spatial video frame data and motion between the frames into account. Due to this edge offered in the case of 3D ConvNets, they are increasingly becoming important in the field of human activity recognition. Thus, in this paper, 3D ConvNets were utilized for training the models in the case of noncontact-based sensors.

The training steps used for building the network are as follows:

- The model was trained on the ImageNet dataset [28]. ImageNet is an extensive image dataset that comprises more than 14 million images and 1000 classes such as animals, sports, and daily human activities. This helped the model to learn basic generic characteristics about the RGB images, such as identifying border, texture, and color.
- From the CNN model trained on the ImageNet dataset, the input layers were modified to take the input as 224 × 224 × 20, where 224 × 224 is the size of the image and 20 is the stack size. Then the model was fine-tuned on the UCF101 fall dataset [20], which comprises 13,320 videos of 101 different human activities. It helped the model to learn the features of generic human actions. To avoid biasing errors, the fall video frames were reduced in order to contain falls only. 3D ConvNet was used here for training the models.
- In the final step, the obtained model was fine-tuned over the UP-Fall dataset.

The following experiments were done to decide the configuration to be used for the training phase.

*(1) Learning Rate*. It is a very important hyperparameter that decides how the model weights are updated during the training. Deciding on a proper learning rate is very important as very small learning rates can make the training too slow, and higher values can make it too fast to converge the model optimally. It was observed from a series of experiments that the network provided the best results when the learning rate was between 10^−3^ and 10^−4^. Choosing higher or lower values affected the accuracy, as lower learning made the system too slow and higher learning made too big steps which made it far away from achieving proper optimization in those cases.

*(2) Minibatch Size*. Li et al. [29], in their study, showed that using minibatch size properly with proper experimentation yields better results than the standard gradient descent. Minibatch gradient is used to split the dataset into batches, and then the training of the network is done in batches; that is, model weights are updated in the batches. Due to a greater number of updates done using minibatch, a good convergence is obtained by using minibatch gradient descent. We tried batch sizes in powers of 2. It was observed through a series of an experiment that a batch size of 1024 was giving the best results.

*(3) Number of Epochs*. Epoch is the number of times the algorithm passes through the training dataset. It is an important parameter for balancing between the time taken for training (with greater number of epochs, higher computation is required) and the saturation obtained. The number of epochs used was 500 in all the experiments, as proper saturation was seen to be obtained within the 500 epochs.

One more important step in the training process is batch normalization. Loffe et al. [30], in their research, explained that making the batch normalization method part of the minibatch training process can make the training process much faster. It was further shown that it eliminates the need for using dropout rates to a great extent. The activation function ReLU (Rectified Linear Unit) was used in the experiments with batch normalization to avoid overfitting and faster training.

**Figure 8:**
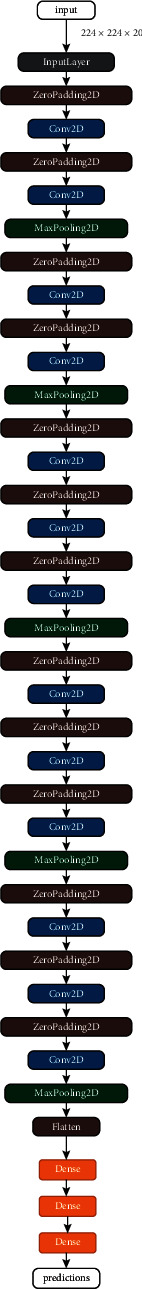
VGG-16 architecture: padding layers in brown, convolution layer in blue, pooling layers in green, and dense layers in orange.

**Figure 9:**
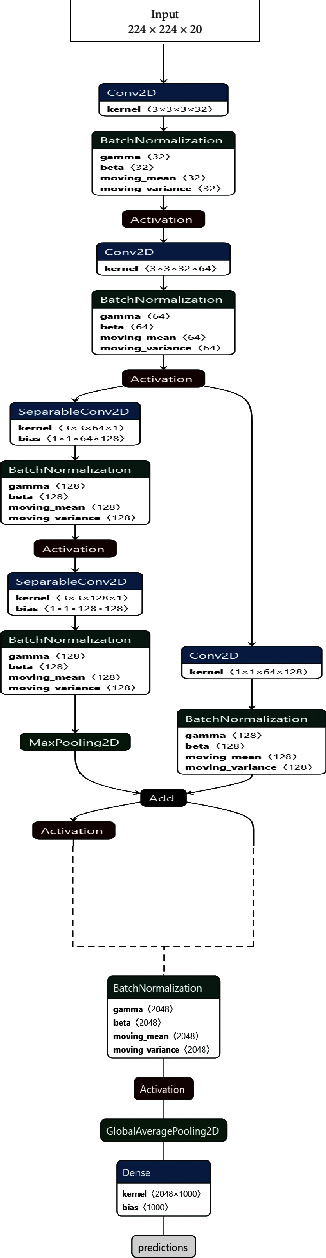
Overview of the Xception architecture implemented. Due to large number of layers present, some of the layers from the start and end are shown here.

**Figure 10:**
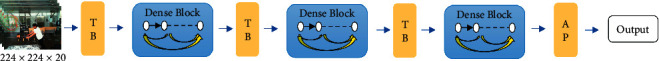
Overview of the DenseNet architecture. T.B. is transition block, and A.P. is average pooling and connected block.

After fixing the hyperparameters, three different architectures backbones experimented with the 3D ConvNet to fine-tune on UP-Fall dataset. These three were chosen to consider the different types of diverse combinations. In two phases, the training of the model was done. Firstly, RGB images were trained on the architectures in two parts, initially on the frontal view and then on the lateral view. The optical flow images were trained similarly. The architecture used was as follows:

1. VGG-16 [31]. It is a very popular CNN architecture that is widely used for classification purposes. It consists of 16 layers for training. It uses 3 × 3 convolutional kernels and 2 × 2 pooling kernels. The consistent arrangement of these layers is followed throughout the architecture. The input shape of 224 × 224 was used in the architecture.  Figure 8 describes the layers of the VGG-16 architecture used.
2. Xception [32]. Xception is a CNN architecture that is 71 layers deep. With modified depth-wise separable convolution, it is a modification of Inception v3 with a parameter that is almost equal to Inception v3.  Figure 9 gives an overview of the Xception architecture implemented by showing some of the layers presented.
3. DenseNet [33]. DenseNet is a CNN architecture in which the layers are densely concentrated, which makes its training time somewhat more than other architectures, but, at the same time, it leads to better convergence of the model. There are many different versions of DenseNet, which vary by the difference in the layer depth. In the experimentation, DenseNet201 with 201 layers was used. The major advantage of using DenseNet as compared to other architectures is that it is more efficient than the other models in capturing low-level features of the images. The overview of the DenseNet architecture can be seen in Figure 10.

## 5. Results and Analysis

For evaluating the performance of models, developed accuracy, sensitivity, specificity, precision, and *F*1-Score were used. Accuracy defines the correctness of the classification model. Sensitivity and specificity define the ability of the system to detect the falls and nonfall events, respectively. Precision predicts the rate of happening of positive events. *F*1-Score is another measure of accuracy focusing on precision and sensitivity.

Equations (1)–(5) define the formulas used to calculate them. Here, F.P. denotes False Positive, F.N. denotes False Negative, T.P. denotes True Positive, and T.N. denotes True Negative. These values are defined as follows:

(1) $$\begin{array}{c} Accuracy=\frac{\left(T.P+T.N\right)}{T.P.+T.N.+F.P.+F.N}\text{,} \end{array}$$

(2) $$\begin{array}{c} Sensitivity=\frac{\;T.P.}{\left(T.P.+F.N.\right)}\text{,} \end{array}$$

(3) $$\begin{array}{c} Specificity=\frac{\;T.N.}{\left(T.N.+F.P\right)}\text{,} \end{array}$$

(4) $$\begin{array}{c} Precision=\frac{T.P.}{\left(T.P.+F.P\right)}\text{,} \end{array}$$

(5) $$\begin{array}{c} F1-Score=2.\frac{\left(Precision.Sensitivity\right)}{\left(Precision+Sensitivity\right)}\text{.} \end{array}$$

The models trained on similar datasets were compared based on these parameters. All the experiments related to CNN architectures were performed on Python v3.7.3 with Keras v2.2 [34] framework using TensorFlow as backend, and ML methods were trained using sklearn library [35]. The hardware used for the experimentation includes 2 Nvidia GTX 1080 Ti graphic cards with 32 GB physical RAM and Intel i7 3.6 GHz processor.

### 5.1. Using Contact-Based Sensors

#### 5.1.1. Using ML Methods

The 3-axial data obtained from the accelerometer fitted on the right wrist of the subject was used for training the model on 3 machine learning methods, which were Support Vector Machine, Random Forest, and Multilayer Perceptron. The data obtained from accelerometers captured acceleration along the *x*-axis, *y*-axis, and *z*-axis in continuous time frames. The data was further divided into training set and test set in the ratio of 70 : 30.

The results obtained from the three algorithms are summarized in Table 3. It can be seen that the RF method performed the best among all the machine learning methods on all the performance metrics. The results also suggest that ML methods can provide significant efficiency on the sequential data stream provided by the accelerometers. To improve on the performance, we further tried the 1D CNN method.

#### 5.1.2. Using 1D CNN Method

Convolutional neural network methods are very good at training sequential streams of data; here 1D CNN-based method was implemented. In 1D CNN, data streams are passed after converting them into 1D arrays and passing them through multiple 1-dimensional layers for training. The training was performed for 500 epochs, with a decent gradient optimizer with a learning rate of 10^−4^.

Here the descent gradient optimizer was used with a negative log-likelihood cost function, as shown in the following equation:

(6) $$\begin{array}{c} Loss\left(x\right)=-\log\left(x\right)\text{.} \end{array}$$

**Figure 11:**
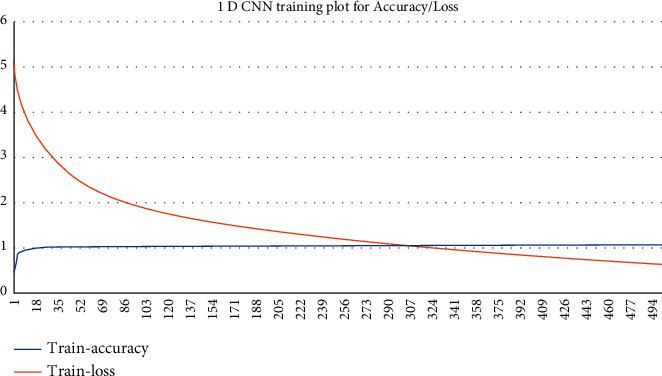
Training accuracy/loss plot obtained in the case of 1D CNN.

Figure 11 shows the accuracy/loss plots obtained in this case. An accuracy of 98.07% was obtained, shown in Table 3. Thus, it was observed to be better than the other implemented ML algorithms.

### 5.2. Using Noncontact-Based Sensors

The model's training was done in two phases through RGB images and optical flow images. RGB images included the frames obtained directly from the camera, while optical flow images were obtained to capture the motion between the consecutive frames. Then, in both cases, the dataset was divided into training set, 80%, and test set, 20%.

The *K* cross-validation approach was used to remove the biasing effect, where *K* refers to the number of different types of groups the data was split into. It is a beneficial technique as it helps get the models variety of combination training and testing datasets. The value of *K* was chosen to be 5 as selecting a higher value of *K* leads to overfitting, and a lower value of *K* is not high enough to remove the biasing effect, so the value of 5 seemed optimal.

#### 5.2.1. Using RGB Cameras

In this experiment, three architectures were trained, namely, VGG-16, Xception, and DenseNet, on lateral and frontal views of the subject. The learning rate was set at 10^−6^, with batch normalization with ReLU as an activation function. The network was then trained for 500 epochs.

**Figure 12:**
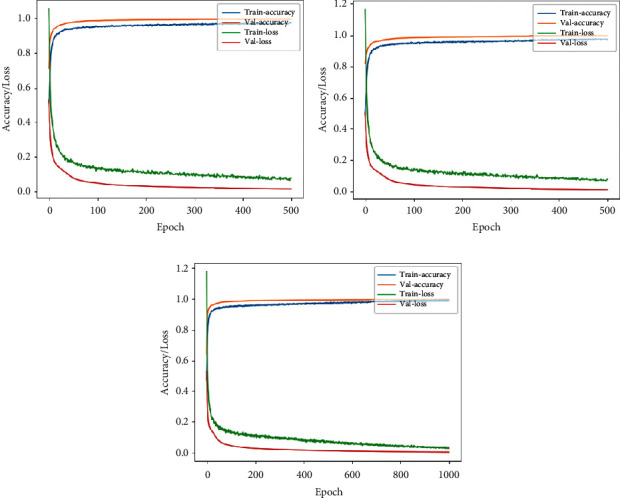
Accuracy/loss training plot for the case of RGB camera recordings in case of lateral view for the three architectures. (a) VGG-16. (b) DenseNet. (c) Xception.

**Figure 13:**
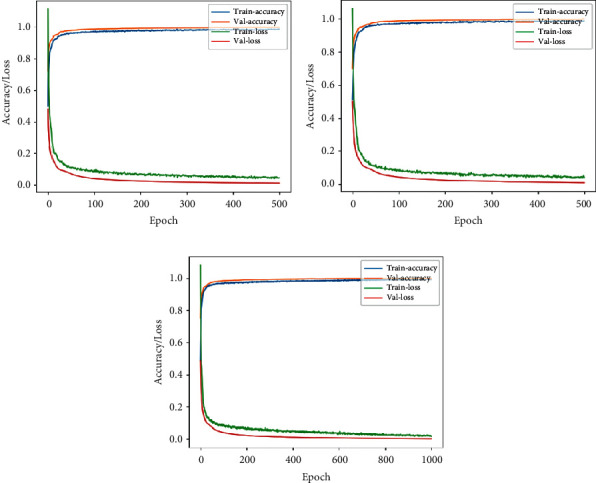
Accuracy/loss training plot for the case of RGB camera recordings in case of frontal view for the three architectures. (a) VGG-16. (b) DenseNet. (c) Xception.

Tables 4 and 5 show the summary of the performances of all the three architectures in lateral and front views, respectively. Figures 12 and 13 highlight the accuracy/loss plots for lateral and frontal views, respectively.

The frontal view was seen to be slightly better than the lateral view; this is because the subject occupies more part of the image in the former case. It can be observed that DenseNet outperformed other architectures with an accuracy of 98.41% in the case of lateral camera recordings and with an accuracy of 99.85% in the case of frontal camera recordings. So, it can be concluded that DenseNet has better performance than the other architectures in the case of fall detection.

When compared with the other vision-based fall detection methods reported in the literature [14, 36], which also implemented CNN-type architectures on UP-Fall detection dataset, as shown in Table 6, it can be seen that the proposed methodology produced results that are comparable with the state-of-the-art methods.

#### 5.2.2. Using Optical Flow Images

Optical flow images are constructed based on the information between the consecutive frames. They capture the motion, if any, between the continuous frames and remove background noises. Tables 7 and 8 show the performances of the architectures in the case of optical flow images of lateral and frontal side views, respectively. They compare the architectures in both the lateral and frontal views based on accuracy, precision, sensitivity, specificity, and *F*1-Score.

The learning rate was kept at 10^−6^, batch normalization with ReLU as activation function was used, and again it was observed and concluded that the DenseNet architecture gave better performance than the other architectures in both the lateral and frontal views. Also, it was observed that the frontal view was giving better performance when compared to the lateral view due to the subject occupying more space in the former case.

**Figure 14:**
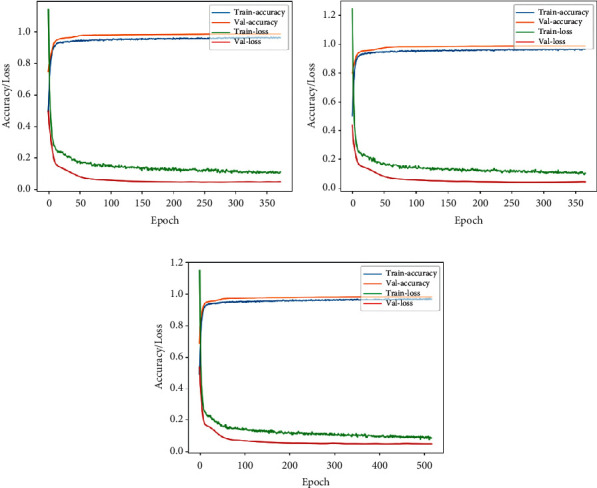
Accuracy/loss training plot for the case of optical flow images in case of lateral view for the three architectures. (a) VGG-16. (b) DenseNet. (c) Xception.

**Figure 15:**
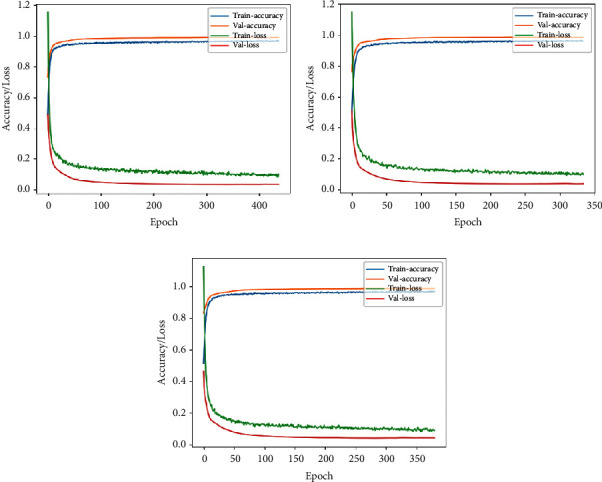
Accuracy/loss training plot for the case of optical flow images in case of frontal view for the three architectures. (a) VGG-16. (b) DenseNet. (c) Xception.

In Figures 14 and 15, training accuracy/loss plots are shown for all three architectures in the case of lateral and frontal views, respectively.

Overall, two approaches based on the contact- and noncontact-based sensors data were attempted to detect falls. Analysis of the results suggests that the 1D CNN method, with an accuracy of 98.07%, outperformed the traditional machine learning methods in the case of contact-based sensors.

It can also be seen that dataset preprocessing steps, which include segmentation and feature extraction, are very important steps for improving the performance of the models by leading to better convergence. Similarly, with an accuracy as high as 99.85% in the case of noncontact sensors, DenseNet outperformed the other architectures and it is shown that very good predictions can be made using camera recordings as well.

The two methods using contact- and noncontact-based sensors discussed above were totally independent. The key advantages of the approaches are discussed here. First, user privacy is a very important concern when going for fall detection based on video recordings. To tackle this, the previous recording can be discarded immediately from the system after processing. In sensor-based detection, only data from the accelerometer is required, so there are not many issues of personal privacy there. For further improving privacy through video camera recording, an optical flow camera-based approach was also discussed, which allows only relevant information. Thus, in both approaches, privacy of an individual can be preserved. Second, in sensor-based fall detection using 1D CNN, the proposed architecture is very efficient in terms of high efficiency at a very low computation cost due to the simplicity of the architecture.

The architectures were implemented on the UP-Fall detection dataset, which includes 17 individuals performing 11 activities. Out of 11 activities, 5 activities were classified as fall, and the other 6 were classified as nonfall. The actors in the dataset were aged 18–24 years. Since the fall activity is only composed of motion, the approaches can be easily transferred to people of all age groups. So, we think these approaches can be extended to apply to real-life conditions as well.

It is also very important to discuss the limitations of the proposed approaches. There are many activities that can be classified into falls. Detecting different falls happening for a very short period can be difficult. For achieving very good efficiency in the case of camera-based detection, the computation cost of the system required will be very high. For real-time fall detection, the system processing speed would be required to be on par with the model processing time as there should not be lag; otherwise, that lag would keep on accumulating to create a huge difference in time and memory requirements.

One important difference between the two approaches is that fall using noncontact-based sensors has low computation cost, and the user has the flexibility of movement. Meanwhile, through camera-based fall detection, the user is required to be inside the frame of the recording. Even falls happening on the edges could be difficult to recognize.

## 6. Conclusions

In this paper, we presented two approaches for fall detection which were using video camera recordings and accelerometer sensors that are commonly found in smartphones. These were done using machine learning and deep learning methods. In the case of contact-based sensors, 1D CNN and machine learning methods were studied. Then, in the case of noncontact-based sensors, 3D ConvNets were implemented utilizing 3 different CNN-based architectures: VGG-16, Xception, and DenseNet.

From the conducted experiments, various things can be concluded. In the case of vision-based sensors, from the three different architectures attempted for implementation, VGG-16 was found to be computationally faster (due to only 16 layers) with good accuracy and comparable with the literature. For further improving on the accuracy, DenseNet is suggested, which performed best among the architectures, but this was obtained at the cost of increasing computation due to a greater number of densely connected layers. In the case of contact-based Sensors, the proposed 1D CNN architectures were shown to outperform ML methods. Both methods have their particular advantages in various conditions. Particularly, sensor-based detection showed various advantages, which include user privacy, no restricted area for movement, and simple architecture.

As can be seen from Table 6, our proposed methodology outperforms the existing architectures with an accuracy of 99.85% as compared to 96.70% of the current state-of-the-art methods. Further improvements in this research should be directed towards embedded board implementation and privacy preservation of the subject by using advanced methods such as compressive sensing and feature encoding.

## Figures and Tables

**Table 1:** Parameters used for ML methods.

Architecture used	Parameters
SVM method	Kernel = “radial basis function”
	*C* = 1
	Gamma = 0.1
	Tolerance = 0.001

RF method	Estimators = 100
	Criterion = “Gini”
	Sample split = 2
	Min. sample leaf = 1
	Max. depth = none

MLP method	Activation = “ReLU”
	Solver = “Adam”
	Max. number of iterations = 200
	Tolerance = 0.0001
	Shuffle = true
	Initial learning rate = 0.001
	Beta1 (exponential rate decay) = 0.9
	Beta2 (exponential rate decay) = 0.999
	Epsilon (numerical stability measure) = 10−8

**Table 2:** Duration of activities and their classification [15].

Sr. no.	Description	Duration (in seconds)	Classification
1.	Falling forward with knees	10	Fall
2.	Falling backward	10	Fall
3.	Falling forward using hands	10	Fall
4.	Falling sideward	10	Fall
5.	Falling sitting on a chair	10	Fall
6.	Standing	60	Nonfall
7.	Walking	60	Nonfall
8.	Sitting	60	Nonfall
9.	Jumping	30	Nonfall
10.	Laying	60	Nonfall
11.	Picking	10	Nonfall

**Table 3:** Summary of results of ML methods implemented.

Architecture used	Accuracy (%)	Precision (%)	Sensitivity (%)	Specificity (%)	*F*1-Score (%)
SVM method	94.00	93.54	93.00	94.00	93.26
RF method	96.875	96.77	96.77	96.96	96.77
MLP method	93.75	93.54	93.54	93.93	93.53
1D CNN	98.30	1	97.14	1	98.55

**Table 4:** Summary of results of architectures implemented in case of RGB images from a lateral view.

Architecture used	Accuracy (%)	Precision (%)	Sensitivity (%)	Specificity (%)	*F*1-Score (%)
VGG-16	97.19	98.25	96.09	98.29	97.16
Xception	98.04	96.90	99.26	96.82	98.07
DenseNet	98.41	98.53	98.29	98.53	98.41

**Table 5:** Summary of results of architectures implemented in case of RGB Images from a frontal view.

Architecture used	Accuracy (%)	Precision (%)	Sensitivity (%)	Specificity (%)	*F*1-Score (%)
VGG-16	96.58	94.26	98.78	94.39	96.65
Xception	97.80	96.44	96.34	94.39	97.02
DenseNet	99.85	99.87	99.82	99.88	99.84

**Table 6:** Comparison with the literature for fall detection from the frontal view.

Proposal	Dataset	Method	Accuracy (%)
Espinosa et al. [36]	UP-Fall	CNN	95.25
Yadav et al. [14]	UP-Fall	CNN	96.70
Proposed method (DenseNet)	UP-Fall	CNN	99.85

**Table 7:** Summary of results of architectures implemented in case of optical flow images from the lateral view.

Architecture used	Accuracy (%)	Precision (%)	Sensitivity (%)	Specificity (%)	*F*1-Score (%)
VGG-16	94.125	90.57	98.5	89.75	94.37
Xception	96.125	93.617	99.00	93.25	96.23
DenseNet	96.00	94.66	97.5	94.5	96.05

**Table 8:** Summary of results of architectures implemented in case of optical flow images from the frontal view.

Architecture used	Accuracy (%)	Precision (%)	Sensitivity (%)	Specificity (%)	*F*1-Score (%)
VGG-16	92.625	95.46	89.50	95.75	92.38
Xception	93.625	96.28	90.75	96.50	93.43
DenseNet	96.00	96.46	95.50	96.50	95.97

## Data Availability

The UP-Fall detection data used to support the findings of this study have been deposited in the UP-Fall repository (https://sites.google.com/up.edu.mx/har-up/).
